# Cross-sectional and longitudinal associations between self-esteem and BMI depends on baseline BMI category in a population-based study

**DOI:** 10.1186/s12889-024-17755-z

**Published:** 2024-01-19

**Authors:** Margaux Robert, Benjamin Allès, Ulrike A. Gisch, Rebecca Shankland, Serge Hercberg, Mathilde Touvier, Christophe Leys, Sandrine Péneau

**Affiliations:** 1 Université Sorbonne Paris Nord and Université Paris Cité, INSERM, INRAE, CNAM, Center of Research in Epidemiology and StatisticS (CRESS), Nutritional Epidemiology Research Team (EREN), F-93017, Bobigny, France; 2 Department of Psychology, Counseling Psychology, University of Potsdam, Karl-Liebknecht-Str. 24-25, 14476 Potsdam, Germany; 3 Institute of Nutritional Science, Department of Nutritional Psychology, Justus Liebig University Giessen, Giessen, Germany; 4 DIPHE Laboratory (Développement, Individu, Processus, Handicap, Education), University Lumière Lyon 2, Lyon, France; 5 Public Health Department, Avicenne Hospital, Assistance Publique-Hôpitaux de Paris (AP-HP), Bobigny, France; 6 Service of Analysis of the Data (SAD), Université Libre de Bruxelles, Bruxelles, Belgium

**Keywords:** Self-esteem, Body mass index, Weight change, Psychological determinants

## Abstract

**Background:**

Some studies have reported associations between self-esteem and weight status, but longitudinal data on adults remain scarce. The aim of this population-based study was to analyze the cross-sectional and longitudinal association between self-esteem and body mass index (BMI) and to investigate whether baseline BMI has an impact on this association.

**Methods:**

In 2016, 29,735 participants aged ≥ 18 years in the NutriNet-Santé cohort completed the Rosenberg Self-Esteem Scale. BMI was self-reported yearly over a 4-year period. Association between self-esteem and BMI was assessed using mixed models and logistic regressions. Analyses were stratified by BMI (categorical) at baseline and adjusted on sociodemographic and lifestyle characteristics.

**Results:**

At baseline, higher self-esteem was associated with higher BMI in normal weight individuals(*p* = 0.32), and with lower BMI in obese class II and III individuals (*p* = 0.13). In addition, higher baseline self-esteem was associated with BMI increase over time in normal weight individuals (*p* = 0.15). Among normal weight individuals, those with higher self-esteem were less likely to show a decrease in their BMI (*p* = 0.005), while no association was observed with BMI increase (*p* = 0.81).

**Discussion:**

Our findings suggest that the association between self-esteem and BMI depends on the initial category of BMI, with a negligible effect of self-esteem.

**Supplementary Information:**

The online version contains supplementary material available at 10.1186/s12889-024-17755-z.

## Introduction

Obesity is recognized as a non-communicable disease, increasing the risk of type 2 diabetes, cardiovascular diseases, certain types of cancer [1], and decreasing life expectancy [2, 3]. The prevalence of obesity worldwide nearly tripled between 1975 and 2019, and continues to grow at a pandemic rate [4]. In France, 17% of adults were obese and 49% were overweight (obesity included) in 2015, with a stable prevalence since 2006 [5]. Overweight and obesity have an important economic impact, as they are estimated to account for 4.9% of health expenditure in France [6], and therefore represent a major public health issue that must be tackled.

Numerous factors are involved in the development of overweight and obesity, such as genetic [1], environment [1] or psychology [7]. Self-esteem is a positive psychological trait referring to an individual’s evaluation of their own worth [8]. Higher levels of self-esteem have been associated with psychological and physical health, such as less anxiety [9], lower risk of coronary heart disease [10] or greater longevity [11]. Higher self-esteem could also be associated with better weight status since it has been associated with greater physical activity [12], healthier eating habits, including a greater adherence to the Mediterranean diet [13] and lower intake of soft drinks [14], and less eating disorders [15]. Although associations between self-esteem on weight has been suggested in the literature [16–20], studies exploring the impact of self-esteem on weight among a general population of adults are still scarce. A cross-sectional study found that self-esteem was negatively associated with body mass index (BMI) in young adults [21]. In addition, greater self-esteem was a predictor of successful weight loss in adults participating in a weight loss reduction program [22–24] or undergoing bariatric surgery [25]. To our knowledge, no study has investigated the potential longitudinal association between self-esteem and weight change in a general adult population. In addition study should consider potential confounders such as socio-demographic and lifestyle factors to minimize biases [26].

In addition, it is possible that the associations between self-esteem and BMI are different depending on the class of BMI in which the participant is in. Given the potential deleterious impact of their weight on physical health [1, 27], and the general social pressure to be thin, individuals with overweight and obesity are often pressured to lose weight. Individuals with higher self-esteem might have better coping skills that allow them to control their weight and at the same time be less impacted by pressure to lose weight. To a lesser extent, normal weight individuals may also attempt to fit to social norms of body shape and thus be more likely to diet. Individuals with higher self-esteem might be less influenced by these social norms, while they also may have more occasion to share meals with friends or family due to their higher perceived social skills [11], which can result in an increase in energy intake [28].

Our aim was to investigate the associations between self-esteem and BMI at baseline, and with BMI change over four years in a large sample of individuals of the NutriNet-Santé cohort, controlling for sociodemographic and lifestyle characteristics. In addition, we investigated whether baseline BMI would modify the associations between self-esteem and BMI (at baseline and change).

## Methods

### Study population and design

This study was conducted as part of the NutriNet-Santé Study, an ongoing web-based prospective cohort of French adult volunteers, launched in 2009. Its aims are to explore the relationships between nutrition and health, as well as the determinants of eating behavior and nutritional status. The rational, design and methods of the study have been described elsewhere [29]. At inclusion and every year after inclusion, volunteers complete several web-based questionnaires to assess their diet, anthropometric measures, lifestyles characteristics, socioeconomic conditions, physical activity and health status. This set of web-based questionnaires has been validated against traditional methods [30–32]. Complementary questionnaires related to determinants of eating behaviors, nutritional status and specific health-related aspects are sent to participants each month.

The NutriNet-Santé study was conducted in accordance with the Declaration of Helsinki, and all procedures were approved by the Institutional Review Board of the French Institute for Health and Medical Research (IRB Inserm n◦ 0000388FWA00005831) and the Commission Nationale Informatique et Libertés (CNIL n◦ 908,450 and n◦ 909,216). Electronic informed consent was obtained from all participants. The study was registered at clinicaltrials.gov as #NCT03335644 (08/11/2017).

### Assessment of self-esteem

Self-esteem was measured with the French version [33] of the Rosenberg Self-Esteem Scale (R-SES) [8] between October and December 2016. This self-report questionnaire was optional. The R-SES is composed of 10 items, 5 positively worded (e.g. “I am able to do things as well as most other people”) and 5 negatively worded (e.g. “I feel I do not have much to be proud of”). Each item is scored on a 4-point Likert Scale ranging from 1 (strongly agree) to 4 (strongly disagree). After reversing the scoring of negatively worded items, items scores were summed then divided by the number of items. The final score was ranging from 1 (low self-esteem) to 4 (high self-esteem). The scale displayed good internal consistency (Cronbach’s α = 0.88).

### Assessment of BMI

Self-reported height and weight were collected at least once a year using a web-based questionnaire. This questionnaire has been validated against traditional paper-and-pencil questionnaire [31] and against measured weight and height by trained staff [34]. BMI was computed as the ratio of weight (kg) to squared height (m^2^). We used all available BMI data from the completion of the R-SES (baseline) to the last available data in the NutriNet-Santé cohort, representing up to four years of follow-up. The median follow-up time was 22 months. BMI was classified according to the WHO references values [1] as follows: normal weight (BMI: 18.5–24.9 kg/m²), overweight (excluding obesity) (BMI: 25.0–29.9 kg/m^2^), obese class I (BMI: 30.0–34.9 kg/m²), obese class II & III (BMI ≥ 35.0 kg/m²). Delta BMI was calculated as the difference between the last and the first available data and categorized as decrease (Delta BMI < 0.0 kg/m²), no change (Delta BMI = 0.0 kg/m²) and increase (Delta BMI > 0.0 kg/m²) in BMI.

### Covariates

Potential confounders of the relationship between self-esteem and BMI were collected and the latest data available prior to the completion of the R-SES (baseline) were retained. These data are provided yearly by the participants and included age (years), sex (men, women), educational level (primary, secondary, undergraduate, and postgraduate), occupational status (unemployed, student, self-employed and farmer, employee and manual worker, intermediate profession, managerial staff and intellectual profession, and retired), monthly income per household unit, smoking status (current smoker, former smoker, and never smoker), physical activity, energy intake (including alcohol) and depressive symptomatology. Monthly income per household unit was calculated using information about income and household composition. The number of people in the household was converted into a number of consumption units (CU) according to the OECD (Organization for Economic Cooperation and Development) equivalence scale: one CU is attributed for the first adult in the household, 0.5 for other persons aged 14 or older and 0.3 for children under 14 [35]. Categories of monthly income were defined as follows: < 1,200; 1,200-1,799; 1,800-2,299; 2,300-2,699; 2,700-3,699; and ≥ 3,700 euros per household unit as well as “unwilling to answer”. Smoking status was assessed by asking participants whether they smoked daily (at least one cigarette, cigar or pipe per day), occasionally (less than one cigarette, cigar or pipe per day), were non-smokers but had previously smoked, or were non-smokers and had never smoked. Physical activity was assessed with the short form of the French version of the International Physical Activity Questionnaire [36]. Weekly energy expenditure, expressed in Metabolic Equivalent of Task in minutes per week (MET in minutes/week), was estimated and three levels of physical activity were defined: low (< 30 min/day), moderate (30–59 min/day), and high (≥ 60 min/day). Energy intake (kcal) was assessed with a set of three 24-hr-dietary records that participants are asked to complete every 6 months. Participants reported all foods and beverages consumed in a day, using standard measurements and/or validated photographs when reporting portion sizes [37]. Mean daily food intake (in grams per day) was weighted according to the day of the week (weekday or weekend). Nutrient and energy intakes were estimated by using the published NutriNet-Santé food composition Table [38]. The modified French National Nutrition and Health Program Guideline Score (mPNNS-GS), which is an a priori nutritional diet quality score, based on adherence to the French food-based dietary guidelines that were in place at the time of the R-SES measurement [39]. The score comprises 12 components: 8 refer to food portion recommendations (regarding fruit and vegetables, starchy food, whole-grain foods, dairy products, meat, eggs and fish, seafood, vegetable fats, and water and soda), and 4 refer to moderation of nutrients or foods (regarding salt, sugar, added fat, and alcohol). Points are deducted for overconsumption of salt and added sugars from sweetened food, as well as when energy intake exceeds the energy requirement by more than 5%, as assessed by the individual’s activity level and the basal metabolic rate (calculated using the Schofield Eq.  [40]). Depressive symptomatology was assessed with the French version [41] of the Center for Epidemiology Studies-Depression (CES-D) scale [42], a 20-item questionnaire rated on a 4-point scale, with higher scores reflecting higher depressive symptomatology. Participants were classified according to the presence of depressive symptomatology (no vs. yes) using the cut-off of 16 [42]. In our sample, the CES-D showed good internal consistency (Cronbach’s α = 0.91). Trait Anxiety was assessed with the French version of the State-Trait Anxiety Inventory (STAI-T) [43] a 20-item questionnaire rated on a 4-point scale. The STAI-T total score ranged from 20 (lower anxiety) to 80 (higher anxiety).

### Statistical analyses

A total of 32,785 participants completed the optional R-SES among the 120,559 participants who received it. Among these participants, 39 were excluded because of acquiescence bias (agreeing to all question without consideration of the reverse items), 1,571 were excluded because of missing data on weight or height and 1,440 participants were excluded because they were underweight (BMI < 18.5 kg/m²), leading to a final sample of 29,735 participants.

We used Student’s t-test and Chi-squared test to compare included with excluded participants, as appropriate. Characteristics of the sample according to baseline BMI was compared using linear regression for continuous variables and analysis of variance (ANOVA) for categorical variables.

We used linear mixed models with random effect to assess the association between self-esteem at baseline (independent variable) and repeated measures of BMI (dependent variables). All BMI measures assessed during the 4-year window were used in a single model. Participants with underweight (BMI < 18.5 kg/m²) at baseline were excluded from the analyses to meet the assumption of linearity in the models. Self-esteem score and time were included as fixed effect, and subject and time were included as random effects. Time was calculated as the difference (in year) between the first anthropometric measure and follow-up points. The β-coefficients for self-esteem score represented the cross-sectional association between self-esteem at baseline and BMI at baseline. The β-coefficients for time represented the mean changes of BMI over time. The β-coefficients for the self-esteem score x time interaction represented the longitudinal association between self-esteem at baseline and the changes of BMI over time. We used multinomial logistic regression models to assess the longitudinal association between self-esteem at baseline (independent variable) and categories of delta BMI (dependent variable). The strength of associations was determined by β-coefficient for linear mixed models, odds ratio (ORs) for logistic regression, and 95% confidence intervals (95% CI). We adjusted our analyses for factors likely to have an impact on our independent and dependent variables. We chose these confounding factors based on our hypotheses and because they have been identified as such in the literature [17, 44–46]. Interactions between self-esteem and BMI categories at baseline and between self-esteem and sex were tested, with BMI as the dependent variable. Variables and interactions that reached *p* < 0.15 in univariable models were further combined in a multivariable linear regression model [47]. Because of the significant interactions of self-esteem with BMI categories, all analyses were stratified by BMI categories.

Models were adjusted as follows: model 1: unadjusted; model 2: adjusted for age, sex, educational level, occupational status, monthly household income, smoking status, physical activity and energy intake. Intermediates models (adjusted for age and sex; and adjusted for age, sex, educational level, occupational status and monthly household income) are presented in the supplemental Tables 1 and 2. Further adjustment on follow-up time was performed when delta BMI was the outcome. Sensitivity analyses with additional adjustment for diet quality (mPNNS-GS), depressive symptomatology (CES-D) and trait anxiety (STAI-T) were performed to assess the robustness of the findings and presented in the supplemental Tables 3 and 4.

Missing data with regard to confounders were handled with multiple imputations by fully conditional specification (20 imputed data set) [48, 49]. All tests were two-sided and *p* < 0.05 was considered statistically significant. Statistical analyses were performed using SAS version 9.4 software (SAS Institute, Inc.).

## Results

### Characteristics of the sample

Compared with excluded participants, the 29,735 included participants were older (55.37 ± 13.69 years for included participants vs. 50.42 ± 15.46 years for excluded participants, *p* < 0.0001), comprised a higher proportion of men (27.37% vs. 15.41%, *p* < 0.0001), of individuals with higher monthly income (≥ 2,700€) (33.34% vs. 24.36%, *p* < 0.0001), of individuals with higher physical activity (38.10% vs. 37.18%, *p* = 0.033), and a lower proportion of never smoker (49.84% vs. 53.61%, *p* < 0.0001). In addition, the level of self-esteem was higher among included participants (3.20 ± 0.46 vs. 3.09 ± 0.50, *p* < 0.0001).

The mean age of our sample was 55.37 ± 13.69 years and most of the participants were women (three out of four).

Table 1 shows the characteristics of the sample according to baseline BMI category. Overall, there was a significant linear trend between every variable analyzed and the categories of BMI (all *p* < 0.0001). Overall, compared to participants with higher BMI, those with lower BMI were more often men, were more often from intermediate or managerial staff and intellectual profession, had more often a high level of education, and a high monthly income per household, were more often never smokers, had more often a high physical activity, had a lower energy intake and had less often depressive symptoms. The median follow-up time was 22 months.

**Table 1:** Individual characteristics of the 29,735 participants of the NutriNet-Santé study (2016), according to baseline BMI category

		All	Normal (18.5–24.9 kg/m²)	Overweight (25.0–29.9 kg/m²)	Obese class I (30.0–34.9 kg/m²)	Obese class II & III (≥ 35.0 kg/m²)	*P* Trend^1^
**N**		29,735	18,809	7,759	2,247	920	
**%**		100	63.26	26.09	7.56	3.09	
**Self-esteem (R-SES)** ^**2**^		3.20 ± 0.46^3^	3.21 ± 0.45	3.21 ± 0.45	3.15 ± 0.48	3.05 ± 0.54	< 0.0001
**Age (years)**		55.37 ± 13.69	53.70 ± 13.99	58.76 ± 12.68	57.67 ± 12.44	55.23 ± 12.31	< 0.0001
**Sex (%)**							< 0.0001
	Men	27.37	23.04	38.01	29.77	20.33	
	Women	72.63	76.96	61.99	70.23	79.67	
**Educational level (%)**							< 0.0001
	Primary	2.23	1.57	3.18	3.65	4.24	
	Secondary	29.48	25.97	33.88	39.48	39.46	
	Undergraduate	31.31	31.69	30.72	30.53	30.54	
	Postgraduate	36.22	40.07	31.38	25.23	25.11	
	Missing data	0.76	0.70	0.84	1.11	0.65	
**Occupational status (%)**							< 0.0001
	Unemployed	7.98	7.91	7.14	9.30	13.37	
	Student	1.03	1.43	0.43	0.13	0.11	
	Self-employed, farmer	1.64	1.77	1.57	1.25	0.76	
	Employee, manual worker	12.29	12.43	10.86	15.04	14.57	
	Intermediate professions	13.78	14.81	11.79	12.19	13.26	
	Managerial staff, intellectual profession	22.00	24.89	17.70	14.69	17.17	
	Retired	39.95	35.25	49.50	46.60	39.24	
	Missing data	1.33	1.51	1.01	0.80	1.52	
**Monthly household income (%)**							< 0.0001
	< 1200 €	8.62	8.03	8.51	11.70	14.02	
	1200–1799 €	19.11	17.87	20.21	22.79	26.09	
	1800–2299 €	14.99	14.70	15.70	15.13	14.57	
	2300–2699 €	10.38	10.38	10.26	10.77	10.43	
	2700–3699 €	18.81	19.91	17.77	15.67	12.83	
	≥ 3700 €	14.52	15.35	14.77	9.43	8.15	
	Unwilling to answer	11.93	11.89	11.52	13.40	12.39	
	Missing data	1.64	1.87	1.26	1.11	1.52	
**Smoking (%)**							< 0.0001
	Current	9.47	9.80	8.70	9.43	9.46	
	Former	40.68	36.98	46.81	48.25	46.19	
	Never	49.84	53.22	44.46	42.32	44.35	
	Missing data	0.01	0.00	0.03	0.00	0.00	
**Physical activity (%)**							< 0.0001
	Low	22.39	20.06	23.22	30.80	42.39	
	Moderate	39.34	40.32	37.97	38.05	34.13	
	High	38.10	39.46	38.66	30.97	23.05	
	Missing data	0.17	0.16	0.15	0.18	0.43	
**Energy intake (Kcal)**		1845.96 ± 483.2	1819.63 ± 459.9	1891.94 ± 511.0	1866.03 ± 516.4	1954.48 ± 576.2	
**Diet quality (mPNNS-GS)**		7.74 ± 1.66	7.75 ± 1.66	7.75 ± 1.64	7.67 ± 1.62	7.41 ± 1.71	< 0.0001
**Depressive symptomatology (CES-D) (%)** ^**4**^							< 0.0001
	No depressive symptom	72.59	73.29	73.98	67.38	59.35	
	Depressive symptom	19.36	17.99	19.15	25.77	33.48	
	Missing data	8.05	8.72	6.87	6.85	7.17	
**Anxiety (STAI-T)** ^**5**^		36.32 ± 10.41	36.31 ± 10.23	35.71 ± 10.31	37.56 ± 11.37	38.96 ± 11.67	< 0.0001
**BMI at baseline (kg/m²)**		24.57 ± 4.39	21.98 ± 1.73	26.99 ± 1.38	31.99 ± 1.38	38.93 ± 3.67	< 0.0001
**Category of delta BMI (%)** ^**6**^							< 0.0001
	Decrease (Delta BMI < 0)	38.87	36.18	42.80	44.05	48.50	
	No change (Delta BMI = 0)	19.11	21.59	15.96	12.23	11.43	
	Increase (Delta BMI > 0)	42.02	42.23	41.24	43.72	40.07	

*Abbreviations: BMI, Body Mass Index; CES-D, Center for Epidemiologic Studies Depression scale; mPNNS-GS, modified French National Nutrition and Health Program Guideline Score; R-SES, Rosenberg Self-Esteem Scale; STAI-T, State-Trait Anxiety Iventory*

^*1*^ *p-trend based on linear regression for continuous variables or ANOVA for categorical variables*

^*2*^ *Score ranges from 1 to 4. The highest score corresponds to the highest self-esteem*

^*3*^ *Mean ± SD, all such values*

^*4*^ *Score ranges from 0 to 60. The highest score corresponds to the highest depressive symptomatology*

^*5*^ *Score ranges from 20 to 80. The highest score corresponds to the highest anxiety*

^*6*^ *Based on 28,374 participants who had more than one BMI value*

### Associations between self-esteem and BMI

Table 2 shows the associations between self-esteem, BMI at baseline and BMI change over time, stratified by baseline BMI category. In individuals with a normal BMI (18.5–24.9 kg/m²), a one-point increase in self-esteem was associated with an increase of 0.058 kg/m² in BMI at baseline *(p* = 0.032) and with an increase of 0.014 kg/m² in BMI per year (*p* = 0.015). In overweight (BMI: 25.0–29.9 kg/m²) or obese class I (BMI: 30–34.9 kg/m²) participants, no association between self-esteem and BMI at baseline or BMI change over time were found. Finally, in participants with obesity class II and III (BMI ≥ 35.0 kg/m²), a one-point increase in self-esteem was associated with a decrease of 0.56 kg/m² in BMI at baseline (*p* = 0.013), while no association was observed with change in BMI over time.

**Table 2:** Association between baseline self-esteem (R-SES) and BMI (baseline and change over time) in 29,735 participants of the NutriNet-Santé Study (2016–2020), according to baseline BMI category

		Model 1^1^			Model 2^2^	
		β-coefficient (95% CI)	*P* Value^3^		β-coefficient (95% CI)	*P* Value^3^
**Normal (18.5–24.9 kg/m²) **(***N* = 18,809)**						
	Self-esteem score, baseline testing^4^	0.129 (0.074, 0.183)	**< 0.0001**		0.058 (0.005, 0.111)	**0.032**
	Time^5^	0.026 (-0.01, 0.062)	0.16		0.181 (0.121, 0.241)	**< 0.0001**
	Self-esteem score x time^6^	0.002 (-0.01, 0.013)	0.78		0.014 (0.003, 0.025)	**0.015**
**Overweight (25.0–29.9 kg/m²) (** *N* ** = 7,759)**						
	Self-esteem score, baseline testing^4^	-0.06 (-0.131, 0.011)	0.098		-0.032 (-0.104, 0.039)	0.38
	Time^5^	-0.013 (-0.095, 0.07)	0.76		0.251 (0.106, 0.395)	**0.0007**
	Self-esteem score x time^6^	0.0001 (-0.025, 0.025)	0.99		0.014 (-0.012, 0.039)	0.30
**Obesity class I (30.0–34.9 kg/m²) (** *N* ** = 2,247)**						
	Self-esteem score, baseline testing^4^	0.0003 (-0.132, 0.132)	0.99		0.027 (-0.108, 0.162)	0.70
	Time^5^	0.163 (-0.031, 0.357)	0.10		0.439 (0.095, 0.784)	**0.013**
	Self-esteem score x time^6^	-0.063 (-0.124, -0.002)	**0.044**		-0.040 (-0.102, 0.023)	0.21
**Obesity class II & III (≥ 35.0 kg/m²) (** *N* ** = 920)**						
	Self-esteem score, baseline testing^4^	-0.803 (-1.238, -0.367)	**0.0003**		-0.562 (-1.006, -0.118)	**0.013**
	Time^5^	-0.400 (-0.856, 0.056)	0.086		-0.240 (-1.153, 0.674)	0.61
	Self-esteem score x time^6^	0.047 (-0.099, 0.194)	0.53		0.018 (-0.135, 0.170)	0.82

*Abbreviation: BMI, Body Mass Index; CI, Confidence Interval; R-SES, Rosenberg Self-Esteem Scale*

^*1*^ *model 1: unadjusted*

^*2*^ *model 2: adjusted on age, gender, educational level, occupational status, monthly household income, smoking status, physical activity and energy intake*

^*3*^ *P value based on linear mixed models with self-esteem as a continuous independent variable*

^*4*^ *The β coefficient for the self-esteem score represents the cross-sectional association between baseline self-esteem and baseline BMI. It corresponds to the BMI variation for an increase of one self-esteem unit (self-esteem score range: 1–4)*

^*5*^ *The β coefficient for time represent the mean evolution of BMI per year*

^*6*^ *The β coefficient for the self-esteem score interaction with time represents the association between baseline self-esteem and the change of BMI over time. It corresponds to the BMI variation per year for the increase of one self-esteem unit (self-esteem score range: 1–4)*

### Associations between self-esteem and delta BMI

Table 3 shows the results of the logistic regression models between self-esteem and delta BMI. In the normal weight (BMI: 18.5–24.9 kg/m²) strata, compared to participants with no BMI change (delta BMI = 0 kg/m²), participants with a one-point increase of self-esteem were less likely to have a decrease in BMI (Delta BMI < 0) (OR (95% CI) = 0.88 (0.80;0.96), *P* = 0.005) over time, while no association was found with an increase in BMI (Delta BMI > 0). In addition, no association between self-esteem and delta BMI was observed for participants with overweight or obesity (BMI ≥ 25.0 kg/m²).

**Table 3:** Association between baseline self-esteem (R-SES) and the difference between the last and first BMI data (Delta BMI) in 28,374 participants of the NutriNet-Santé Study (2016–2020)

		Model 1^1^			Model 2^2^	
		OR (95% CI)	*P* Value^3^		OR (95% CI)	*P* Value^3^
**Normal (18.5–24.9 kg/m²) (** ***N*** ** = 17,968)**						
	Decrease (Delta BMI < 0 kg/m²)	0.90 (0.82, 0.98)	**0.017**		0.88 (0.80, 0.96)	**0.005**
	No change (Delta BMI = 0 kg/m²)	Ref			Ref	
	Increase (Delta BMI > 0 kg/m²)	0.95 (0.87, 1.03)	0.22		0.99 (0.91, 1.08)	0.81
**Overweight (18.5–24.9 kg/m²) (** *N* ** = 7,413)**						
	Decrease (Delta BMI < 0 kg/m²)	0.88 (0.76, 1.03)	0.11		0.88 (0.75, 1.03)	0.10
	No change (Delta BMI = 0 kg/m²)	Ref			Ref	
	Increase (Delta BMI > 0 kg/m²)	0.93 (0.80, 1.08)	0.33		0.98 (0.84, 1.15)	0.85
**Obesity class I (30.0–34.9 kg/m²) (** *N* ** = 2,127)**						
	Decrease (Delta BMI < 0 kg/m²)	1.04 (0.78, 1.38)	0.81		1.08 (0.80, 1.46)	0.60
	No change (Delta BMI = 0 kg/m²)	Ref			Ref	
	Increase (Delta BMI > 0 kg/m²)	0.95 (0.71, 1.27)	0.73		1.05 (0.77, 1.41)	0.76
**Obesity class II & III (≥ 35.0 kg/m²) (** *N* ** = 866)**						
	Decrease (Delta BMI < 0 kg/m²)	1.09 (0.73, 1.62)	0.68		0.97 (0.64, 1.49)	0.90
	No change (Delta BMI = 0 kg/m²)	Ref			Ref	
	Increase (Delta BMI > 0 kg/m²)	1.06 (0.71, 1.60)	0.77		1.01 (0.66, 1.56)	0.95

*Abbreviation: BMI, Body Mass Index; CI, Confidence Interval; OR, Odds Ratio R-SES, Rosenberg Self-Esteem Scale*

^*1*^ *model 1: unadjusted*

^*2*^ *model 2: adjusted on age, gender, educational level, occupational status, monthly household income, smoking status, physical activity, energy intake and follow-up time*

^*3*^ *P-Value based on multinomial logistic regression with baseline self-esteem as continuous independent variable and delta BMI as a categorical dependent variable*

### Sensitivity analyses

Sensitivity analyses are shown in supplemental Tables 3 and 4. Further adjustment for the mPNNS-GS score did not substantially change the results as the associations between self-esteem and BMI remained significant for participants with a normal BMI (18.5–24.9) (cross sectional and longitudinal associations) and participants with obesity class II and III (≥ 35 kg/m²) (cross sectional associations). Further adjustment for depressive symptomatology and anxiety showed similar results. The only difference observed was that the association between self-esteem and baseline BMI in normal weight participants became non-significant with both depressive symptomatology and anxiety (*p* > 0.05).

## Discussion

This population-based study assessed the cross-sectional and longitudinal associations between self-esteem and BMI according to baseline BMI. In the group of individuals with a normal BMI range, higher self-esteem was associated with higher BMI at baseline and with an increase in BMI over time. Further analyses investigating this association between self-esteem and delta BMI suggested that this association corresponds in fact to less weight loss over time, rather than weight gain. In participants with obesity class II and III, higher self-esteem was associated with lower BMI at baseline while no association was found with BMI change over time. Finally, in individuals with overweight and obesity class I, no association between self-esteem and BMI was found.

### Individuals with normal range BMI at baseline

In our study, among participants of normal range BMI, higher self-esteem was associated with a higher BMI status at baseline and a greater BMI gain, which would be due to less weight loss over time. These results contrasted with previous studies, conducted among 450 pre-university student aged 16–19 years [21] and among 1157 children aged 7 years [19] with various weight status, that showed a negative association between self-esteem and weight status. Differences in weight status range might have led to these differences between studies. Our results could suggest that individuals with higher self-esteem had a lower tendency to attempt weight loss during the follow-up period. They might engage in fewer dieting behaviors due to their greater body satisfaction [50–52], which has been shown to be inversely related to dieting behavior [53, 54] and weight loss attempt [55]. This potential interpretation should be nuanced by the fact that restrictive diet on the long term lead to long term weight gain [56]. In addition, participants within a normal range of BMI may experience less social pressure to be thin and lose weight. They may also feel less concerned about their diet and weight given that, in general, they have a higher level of body satisfaction [51, 52, 57].

Some other hypotheses can be suggested to explain the positive association between self-esteem and BMI change. Meals in France have an important convivial dimension, since they are often shared with others [58] and are seen as a conviviality and pleasurable moment [58]. Individuals with high self-esteem tend to have higher perceived social skills [11] and may therefore have more occasions to share convivial meals in which they would favor hedonic non-healthy high caloric food [59].

Finally, it is important to note that our results suggest a limited clinical impact of self-esteem on BMI in participants with a normal baseline BMI. The longitudinal association, although significant, was rather negligible, with an increase of 1 point in self-esteem (range: 1–4) associated with an increase in BMI of only 0.014 kg/m² per year. This result, together with the average BMI observed in normal-weight participants (21.98 kg/m² ± 1.73) suggests that participants overall are likely to remain in the BMI class they were in at baseline, underlining the relatively limited effect of self-esteem on BMI. These results are consistent with a previous study conducted among 14 year old high school female students (*N* = 242) showing that self-esteem did not predict residual gain in weight over the 2 years of follow-up [60].

### Individuals with obesity class II or III at baseline

In participants with obesity class II and III, self-esteem was negatively associated with BMI at baseline. To the best of our knowledge, no other studies examined the association between self-esteem and BMI in individuals with obesity class II or III. We hypothesize that the personality of individuals with higher self-esteem could have a specific influence in individuals of this BMI range. Individuals with higher self-esteem have been shown to be more emotionally stable, extraverted, conscientious and somewhat agreeable and open to experience [61], which can in turn be associated with healthier dietary behavior. Greater openness, conscientiousness and emotional stability have been associated with higher intake of heathy food groups such as plant-based food (e.g., fruits and vegetables, legumes) and fish [62], and with greater conscientiousness, emotional stability and lower BMI [62]. Higher self-esteem was also associated with a higher life satisfaction [11], which has been associated to lower weight gain [63]. Finally, individuals with greater self-esteem have been less likely to experience anxiety [9] and depression [64], which are risk factors for changes towards unhealthy eating behaviors [65, 66] and weight gain [67]. Consistently, results of the sensitivity analysis showed that controlling for depressive symptomatology weakened the cross-sectional association between self-esteem and BMI at baseline.

Although cross-sectional analyses indicated a significant negative association, our longitudinal analyses did not confirm these results suggesting that self-esteem did not influence weight change over time in obese individuals. By contrast, other data in the literature indicated that self-esteem was a predictor of successful weight loss in obese adults participating to a weight loss intervention [22] or undergoing bariatric surgery [25]. Methodological limits may also have led to these non-significant results including a relatively short follow up time, and the limited BMI variability within BMI’s strata. Another explanation is that the association between self-esteem and BMI could be inverse in this group (i.e. an impact of BMI on self-esteem), as suggested by previous longitudinal studies [16, 68]. This could be the consequence of greater stigmatization and lower physical activity observed in obese individuals [69, 70], which can lead to changes in self-esteem [70].

### Individuals with overweight or obesity class I at baseline

The longitudinal significant inverse association between self-esteem and BMI observed in the raw model became non-significant after adjustment on age and sex, suggesting a confounding bias of these demographic variables. In the adjusted model, both cross-sectional and longitudinal association between self-esteem and BMI were non-significant, in contrast with a previous study, conducted in adults with overweight and obesity, in which self-esteem was a predictor of weight loss [22]. The absence of a cross-sectional association in this group contrasts with data on individuals with class I and II obesity. This could be due to an intermediate behavior in this group, between participants in the normal range, for which we showed a positive association, and participants obesity class II and III, for which we showed a negative cross-sectional association. This group might also be less exposed to stimuli mediating the association between self-esteem and BMI, such as less body satisfaction or less social skills, compared with participants in the normal BMI group. The absence of longitudinal association is nevertheless consistent with our findings in the obesity class I and II group, for whom no longitudinal association were found either.

### Strengths and limitations

Strengths of this study include its prospective design and its large sample size including participants with various socio-demographic characteristics and nutritional status, which allows the use of multiple covariates to adjust for potential confounding factors. However, we cannot rule out the possibility that other important confounders were not considered. To our knowledge, only a few studies have previously assessed the association between self-esteem and BMI in an adult sample drawn from the general population. Thus, our study provides new data on these associations, particularly within different BMI classes. The level of self-esteem was determined with the R-SES, which has been validated in French [33] and demonstrated good psychometric properties in our study. However, the self-declared aspect of the questionnaire could have led to reporting bias [53]. The self-reported anthropometric measures could also have led to misclassification. However, standardized clinical measurements in a subsample (*N* = 2,513) of the NutriNet-Santé cohort showed good convergence with self-reported data [71]. Another limitation is the use of BMI alone, which is not considered a sufficient measure of obesity because it blurs the distinction between fat and non-fat mass [72]. Further limitations of our study include the relatively short follow-up time and the stratification scheme on baseline BMI which could have led to a decrease in BMI variability within the strata that would lead to a weakening of the associations. Another limitation is that our study might present a selection bias, consequent to the participants’ recruitment methods, based on volunteering. That implies that our subjects may have high health awareness compared to the global population and may therefore not be representative of the French population. However, we can note that the average BMI in our sample (25.4 ± 3.8 in men and 24.27 ± 4.6 in women) was close to the BMI observed in a representative sample of the French population (25,8 kg/m² (25.5–26.1) in men and 25.7 kg/m² (25,2–26.1) in women) [5].

## Conclusion

This study explored the cross-sectional and longitudinal association between self-esteem and BMI, in a large population-based sample of adult women and men, stratified on baseline BMI. In individuals with normal weight, we found that higher self-esteem was associated with greater BMI at baseline and less decrease in BMI over time, although the strength of the association was weak. In participants with class II and III obesity, higher self-esteem was associated with lower BMI at baseline while there was no association with BMI change over time, which suggest that BMI could influence self-esteem rather than the opposite. In individuals with overweight and class I obesity we found no cross-sectional or longitudinal association between self-esteem and BMI. In summary, the overall association between self-esteem and BMI appears to be relatively weak and depending on baseline BMI category. Further population-based studies are needed to confirm our results, and in particular longitudinal studies with a longer follow-up.

### Electronic supplementary material

Below is the link to the electronic supplementary material.

Supplementary Material 1

## Data Availability

Academic researchers from public institution scan submit a collaboration request including a brief description of the project to Mathilde Touvier at collaboration@etude-nutrinet-sante.fr. All requests will be reviewed by the steering committee of the NutriNet-Santé study. A financial contribution may be requested. If the collaboration is accepted, a data access agreement will be necessary and appropriate authorizations from the competent administrative authorities may be needed. In accordance with existing regulations, no personal data will be accessible.

## Abbreviations

BMI: Body Mass Index
CES-D: Center for Epidemiologic Studies Depression scale
CI: Confidence Interval
CU: Consumption Unit
mPNNS-GS: modified French National Nutrition and Health Program Guideline Score
OR: Odds Ratio
R-SES: Rosenberg Self-Esteem Scale
STAI-T: State-Trait Anxiety Iventory
