# Supplementary material for: Cross-sectional and longitudinal associations between self-esteem and BMI depends on baseline BMI category in a population-based study
Source: BMC Public Health. 2024 Jan 19;24:230. doi: 10.1186/s12889-024-17755-z (PMC10797749; doi:10.1186/s12889-024-17755-z)
Supplement: Supplementary file 1 — Supplementary Material 1 [file 12889_2024_17755_MOESM1_ESM.docx]

**Cross-sectional and longitudinal associations between self-esteem and BMI depends on baseline BMI category in a population-based study**

*Supplemental Material*

**Supplemental 1 |** Association between self-esteem at baseline (R-SES) and BMI (baseline and change over time) in 29,735 participants of the NutriNet-Santé Study (2016-2020), according to baseline BMI category, all models.

|  |  | **Model 1a^1^** | |  | **Model 1b^2^** | |  | **Model 1c^3^** | |  | **Model 2^4^** | |
| --- | --- | --- | --- | --- | --- | --- | --- | --- | --- | --- | --- | --- |
|  |  | **β-coefficient (95% CI)** | **P Value^5^** |  | **β-coefficient (95% CI)** | **P Value^5^** |  | **β-coefficient (95% CI)** | **P Value^5^** |  | **β-coefficient (95% CI)** | **P Value^5^** |
| **Normal (18.5 - 24.9 kg/m²) (N = 18,809)** | |  |  |  |  |  |  |  |  |  |  |  |
|  | Self-esteem score, baseline^6^ | 0.129 (0.074, 0.183) | **<.0001** |  | 0.042 (-0.01, 0.095) | 0.11 |  | 0.054 (0.001, 0.107) | **0.044** |  | 0.058 (0.004, 0.112) | **0.036** |
|  | Time (years) ^7^ | 0.002 (-0.001, 0.005) | 0.16 |  | 0.016 (0.013, 0.019) | **<.0001** |  | 0.144 (0.088, 0.2) | **<.0001** |  | 0.183 (0.121, 0.244) | **<.0001** |
|  | Self-esteem score x time*^8^* | 0.0001 (-0.001, 0.001) | 0.78 |  | 0.001 (0, 0.002) | **0.096** |  | 0.013 (0.002, 0.025) | **0.019** |  | 0.015 (0.003, 0.026) | **0.011** |
| **Overweight (25.0 - 29.9kg/m²) (N = 7,759)** | |  |  |  |  |  |  |  |  |  |  |  |
|  | Self-esteem score, baseline^6^ | -0.060 (-0.131, 0.011) | 0.098 |  | -0.064 (-0.135, 0.007) | 0.079 |  | -0.044 (-0.116, 0.028) | 0.23 |  | -0.036 (-0.11, 0.037) | 0.34 |
|  | Time (years) ^7^ | -0.001 (-0.008, 0.006) | 0.76 |  | 0.02 (0.011, 0.028) | **<.0001** |  | 0.204 (0.069, 0.339) | **0.0030** |  | 0.239 (0.094, 0.384) | **0.0012** |
|  | Self-esteem score x time*^8^* | 0.000 (-0.002, 0.002) | 0.99 |  | 0.001 (-0.001, 0.003) | 0.42 |  | 0.015 (-0.011, 0.041) | 0.25 |  | 0.013 (-0.013, 0.039) | 0.3277 |
| **Obesity class I (30.0 - 34.9 kg/m²) (N = 2,247)** | |  |  |  |  |  |  |  |  |  |  |  |
|  | Self-esteem score, baseline^6^ | 0.0003 (-0.131, 0.132) | 0.99 |  | 0.016 (-0.117, 0.149) | 0.81 |  | 0.026 (-0.109, 0.161) | 0.70 |  | 0.041 (-0.097, 0.18) | 0.56 |
|  | Time (years) ^7^ | 0.014 (-0.003, 0.030) | 0.10 |  | 0.026 (0.007, 0.045) | **0.0073** |  | 0.357 (0.03, 0.683) | **0.032** |  | 0.458 (0.113, 0.803) | **0.0093** |
|  | Self-esteem score x time*^8^* | -0.005 (-0.010, 0.0001) | **0.044** |  | -0.004 (-0.009, 0.001) | 0.10 |  | -0.041 (-0.103, 0.022) | 0.20 |  | -0.041 (-0.104, 0.022) | 0.20 |
| **Obesity class II & III (≥ 35.0kg/m²) (N = 920)** | |  |  |  |  |  |  |  |  |  |  |  |
|  | Self-esteem score, baseline^6^ | -0.802 (-1.238, -0.367) | **0.0003** |  | -0.735 (-1.174, -0.296) | **0.0011** |  | -0.634 (-1.079, -0.188) | **0.0053** |  | -0.497 (-0.96, -0.033) | **0.036** |
|  | Time (years)^7^ | -0.033 (-0.071, 0.005) | 0.086 |  | -0.041 (-0.087, 0.005) | 0.079 |  | -0.327 (-1.181, 0.528) | 0.45 |  | -0.185 (-1.102, 0.733) | 0.69 |
|  | Self-esteem score x time*^8^* | 0.004 (-0.008, 0.016) | 0.53 |  | 0.003 (-0.009, 0.016) | 0.60 |  | 0.027 (-0.125, 0.178) | 0.73 |  | 0.044 (-0.109, 0.196) | 0.57 |

*Abbreviation: BMI, Body Mass Index; CI, Confidence Interval; R-SES, Rosenberg Self-Esteem Scale*

*^1^ model 1a: unadjusted. (model 1 in the main document)*

*^2^ model 1b: adjusted on age and gender.*

*^3^ model 1c: model 1b + occupational status and monthly household income.*

*^4^ model 2: model 1c + smoking status, physical activity and energy intake. (model 2 in the main document)*

*^5^ P value based on linear mixed models with self-esteem as a continuous independent variable.*

*^6^ The β coefficient for the self-esteem score represents the cross-sectional association between baseline self-esteem and baseline BMI. It corresponds to the BMI variation for an increase of one self-esteem unit (self-esteem score range: 1 – 4).*

*^7^ The β coefficient for time represents the mean evolution of BMI per year.*

*^8^The β coefficient for the self-esteem score interaction with time represents the association between baseline self-esteem and the change of BMI over time. It corresponds to the BMI variation per year for the increase of one self-esteem unit (self-esteem score range: 1 – 4).*

**Supplemental 2 |** Association between self-esteem (R-SES) and the difference between the last and first BMI data (Delta BMI) in 28,374 participants of the NutriNet-Santé Study (2016-2020), all models.

|  |  | **Model 1a^1^** | |  | **Model 1b^2^** | |  | **Model 1c^3^** | |  | **Model 2^4^** | |
| --- | --- | --- | --- | --- | --- | --- | --- | --- | --- | --- | --- | --- |
|  |  | **OR (95% CI)** | **P Value^5^** |  | **OR (95% CI)** | **P Value^5^** |  | **OR (95% CI)** | **P Value^5^** |  | **OR (95% CI)** | **P Value^5^** |
| **Normal (18.5 - 24.9 kg/m²) (N = 17,968)** | |  |  |  |  |  |  |  |  |  |  |  |
|  | Decrease (Delta BMI < 0 kg/m²) | 0.90 (0.82, 0.98) | **0.017** |  | 0.88 (0.8, 0.96) | **0.0063** |  | 0.88 (0.8, 0.96) | **0.0045** |  | 0.88 (0.80, 0.96) | **0.005** |
|  | No change (Delta BMI = 0 kg/m²) | Ref | |  | Ref | |  | Ref | |  | Ref | |
|  | Increase (Delta BMI > 0 kg/m²) | 0.95 (0.87, 1.03) | 0.22 |  | 0.99 (0.91, 1.09) | 0.87 |  | 0.98 (0.9, 1.07) | 0.70 |  | 0.99 (0.91, 1.08) | 0.81 |
| **Overweight (25.0 - 29.9kg/m²) (N = 7,413)** | |  |  |  |  |  |  |  |  |  |  |  |
|  | Decrease (Delta BMI < 0 kg/m²) | 0.88 (0.76, 1.03) | 0.11 |  | 0.87 (0.74, 1.02) | 0.081 |  | 0.87 (0.75, 1.02) | 0.086 |  | 0.88 (0.75, 1.03) | 0.10 |
|  | No change (Delta BMI = 0 kg/m²) | Ref | |  | Ref | |  | Ref | |  | Ref | |
|  | Increase (Delta BMI > 0 kg/m²) | 0.93 (0.80, 1.08) | 0.33 |  | 0.96 (0.84, 1.15) | 0.81 |  | 0.98 (0.84, 1.15) | 0.80 |  | 0.98 (0.84, 1.15) | 0.85 |
| **Obesity class I (30.0 - 34.9 kg/m²) (N = 2,127)** | |  |  |  |  |  |  |  |  |  |  |  |
|  | Decrease (Delta BMI < 0 kg/m²) | 1.04 (0.78, 1.38) | 0.81 |  | 1.09 (0.8, 1.47) | 0.59 |  | 1.08 (0.8, 1.46) | 0.62 |  | 1.08 (0.80, 1.46) | 0.60 |
|  | No change (Delta BMI = 0 kg/m²) | Ref | |  | Ref | |  | Ref | |  | Ref | |
|  | Increase (Delta BMI > 0 kg/m²) | 0.95 (0.71, 1.27) | 0.73 |  | 1.08 (0.8, 1.46) | 0.61 |  | 1.04 (0.77, 1.4) | 0.80 |  | 1.05 (0.77, 1.41) | 0.76 |
| **Obesity class II & III (≥ 35.0kg/m²) (N = 866)** | |  |  |  |  |  |  |  |  |  |  |  |
|  | Decrease (Delta BMI < 0 kg/m²) | 1.09 (0.73, 1.62) | 0.68 |  | 1.02 (0.66, 1.58) | 0.92 |  | 0.99 (0.65, 1.51) | 0.97 |  | 0.97 (0.64, 1.49) | 0.90 |
|  | No change (Delta BMI = 0 kg/m²) | Ref | |  | Ref | |  | Ref | |  | Ref | |
|  | Increase (Delta BMI > 0 kg/m²) | 1.06 (0.71, 1.60) | 0.77 |  | 1.05 (0.65, 1.56) | 0.96 |  | 1.04 (0.68, 1.59) | 0.86 |  | 1.01 (0.66, 1.56) | 0.95 |

*Abbreviation: BMI, Body Mass Index; CI, Confidence Interval; OR, Odds Ratio R-SES, Rosenberg Self-Esteem Scale*

*^1^ model 1a: unadjusted. (model 1 in the main document)*

*^2^ model 1b: adjusted on age and gender.*

*^3^ model 1c: model 1b + occupational status and monthly household income.*

*^4^ model 2: model 1c + smoking status, physical activity and energy intake. (model 2 in the main document)*

*^5^ P-Value based on multinomial logistic regression with baseline self-esteem as a continuous independent variable and delta BMI* *as a categorical dependent variable.*

**Supplemental 3 |** Association between self-esteem at baseline (R-SES) and BMI (baseline and change over time) in 29,735 participants of the NutriNet-Santé Study (2016-2020), according to baseline BMI category, sensitivity analyses.

|  |  | **Model 3^1^** | |  | **Model 4^2^** | |  | **Model 5^3^** | |
| --- | --- | --- | --- | --- | --- | --- | --- | --- | --- |
|  |  | **β-coefficient (95% CI)** | **P Value^4^** |  | **β-coefficient (95% CI)** | **P Value^4^** |  | **β-coefficient (95% CI)** | **P Value^4^** |
| **Normal (18.5 - 24.9 kg/m²) (N = 18,809)** | |  |  |  |  |  |  |  |  |
|  | Self-esteem score, baseline^5^ | 0.052 (-0.005, 0.108) | 0.074 |  | 0.061 (-0.001, 0.123) | 0.054 |  | 0.058 (0.005, 0.111) | **0.031** |
|  | Time (years)^6^ | 0.149 (0.069, 0.229) | **0.0003** |  | 0.015 (0.010, 0.021) | **<.0001** |  | 0.189 (0.124, 0.254) | **<.0001** |
|  | Self-esteem score x time^7^ | 0.019 (0.005, 0.032) | **0.0068** |  | 0.002 (0.001, 0.003) | **0.0005** |  | 0.014 (0.003, 0.025) | **0.014** |
| **Overweight (25.0 - 29.9kg/m²) (N = 7,759)** | |  |  |  |  |  |  |  |  |
|  | Self-esteem score, baseline^5^ | -0.027 (-0.108, 0.053) | 0.50 |  | 0.013 (-0.071, 0.097) | 0.76 |  | -0.032 (-0.104, 0.04) | 0.38 |
|  | Time (years)^6^ | 0.212 (0.033, 0.391) | **0.021** |  | 0.021 (0.008, 0.034) | **0.0019** |  | 0.252 (0.100, 0.404) | **0.0012** |
|  | Self-esteem score x time^7^ | 0.020 (-0.011, 0.050) | 0.21 |  | 0.001 (-0.001, 0.004) | 0.29 |  | 0.014 (-0.011, 0.04) | 0.28 |
| **Obesity class I (30.0 - 34.9 kg/m²) (N = 2,247)** | |  |  |  |  |  |  |  |  |
|  | Self-esteem score, baseline^5^ | 0.064 (-0.095, 0.223) | 0.43 |  | 0.098 (-0.063, 0.259) | 0.23 |  | 0.027 (-0.108, 0.162) | 0.70 |
|  | Time (years)^6^ | 0.469 (0.020, 0.919) | **0.041** |  | 0.031 (0.000, 0.063) | **0.049** |  | 0.390 (0.016, 0.764) | **0.041** |
|  | Self-esteem score x time^7^ | -0.048 (-0.126, 0.030) | 0.23 |  | -0.002 (-0.008, 0.004) | 0.53 |  | -0.040 (-0.103, 0.022) | 0.20 |
| **Obesity class II & III (≥ 35.0kg/m²) (N = 920)** | |  |  |  |  |  |  |  |  |
|  | Self-esteem score, baseline^5^ | -0.608 (-1.107, -0.109) | **0.017** |  | -0.459 (-0.993, 0.076) | 0.093 |  | -0.569 (-1.013, -0.124) | **0.012** |
|  | Time (years)^6^ | -0.571 (-1.774, 0.632) | 0.35 |  | -0.050 (-0.135, 0.035) | 0.25 |  | 0.011 (-0.921, 0.944) | 0.98 |
|  | Self-esteem score x time^7^ | 0.074 (-0.117, 0.265) | 0.45 |  | -0.004 (-0.019, 0.012) | 0.63 |  | 0.015 (-0.137, 0.168) | 0.84 |

*Abbreviation: BMI, Body Mass Index; CI, Confidence Interval; R-SES, Rosenberg Self-Esteem Scale.*

*^1^ model 3: adjusted on age, gender, educational level, occupational status, monthly household income, smoking status, physical activity, energy intake and diet quality (mPNNS-GS,* *modified French National Nutrition and Health Program Guideline Score).*

*^2^ model 4: adjusted on age, gender, educational level, occupational status, monthly household income, smoking status, physical activity, energy intake and depressive symptomatology (CES-D, Center for Epidemiology Studies-Depression).*

*^3^ model 5: adjusted on age, gender, educational level, occupational status, monthly household income, smoking status, physical activity, energy intake and anxiety (STAI-T, State--Trait Anxiety Inventory).*

*^4^ P value based on linear mixed models with self-esteem as a continuous independent variable.*

*^5^ The β coefficient for the self-esteem score represents the cross-sectional association between baseline self-esteem and baseline BMI. It corresponds to the BMI variation for an increase of one self-esteem unit (self-esteem score range: 1 – 4).*

*^6^ The β coefficient for time represents the mean evolution of BMI per year.*

*^7^The β coefficient for the self-esteem score interaction with time represents the association between baseline self-esteem and the change of BMI over time. It corresponds to the BMI variation per year for the increase of one self-esteem unit (self-esteem score range: 1 – 4).*

**Supplemental 4 |** Association between self-esteem (R-SES) and the difference between the last and first BMI data (Delta BMI) in 28,374 participants of the NutriNet-Santé Study (2016-2020), sensitivity analyses.

|  |  | **Model 3^1^** | |  | **Model 4^2^** | |  | **Model 5^3^** | |
| --- | --- | --- | --- | --- | --- | --- | --- | --- | --- |
|  |  | **OR (95% CI)** | ***P-Value^4^*** |  | **OR (95% CI)** | ***P-Value^4^*** |  | **OR (95% CI)** | ***P-Value^4^*** |
| **Normal (18.5 - 24.9 kg/m²) (N = 17,968)** | |  |  |  |  |  |  |  |  |
|  | Decrease (Delta BMI < 0 kg/m²) | 0.88 (0.8, 0.96) | **0.0049** |  | 0.9 (0.82, 0.99) | **0.036** |  | 0.94 (0.84, 1.06) | 0.31 |
|  | No change (Delta BMI = 0 kg/m²) | Ref | |  | Ref | |  | Ref | |
|  | Increase (Delta BMI > 0 kg/m²) | 0.99 (0.91, 1.08) | 0.84 |  | 1.02 (0.93, 1.12) | 0.66 |  | 1.04 (0.93, 1.16) | 0.47 |
| **Overweight (25.0 - 29.9kg/m²) (N = 7,413)** | |  |  |  |  |  |  |  |  |
|  | Decrease (Delta BMI < 0 kg/m²) | 0.88 (0.75, 1.03) | 0.11 |  | 0.9 (0.76, 1.07) | 0.24 |  | 0.94 (0.78, 1.14) | 0.51 |
|  | No change (Delta BMI = 0 kg/m²) | Ref | |  | Ref | |  | Ref | |
|  | Increase (Delta BMI > 0 kg/m²) | 0.98 (0.84, 1.15) | 0.84 |  | 1.04 (0.88, 1.23) | 0.65 |  | 1.08 (0.89, 1.32) | 0.43 |
| **Obesity class I (30.0 - 34.9 kg/m²) (N = 2,127)** | |  |  |  |  |  |  |  |  |
|  | Decrease (Delta BMI < 0 kg/m²) | 1.08 (0.80, 1.46) | 0.61 |  | 1.03 (0.74, 1.44) | 0.86 |  | 1.21 (0.82, 1.77) | 0.34 |
|  | No change (Delta BMI = 0 kg/m²) | Ref | |  | Ref | |  | Ref | |
|  | Increase (Delta BMI > 0 kg/m²) | 1.04 (0.77, 1.41) | 0.78 |  | 1.02 (0.73, 1.42) | 0.93 |  | 1.05 (0.71, 1.55) | 0.80 |
| **Obesity class II & III (≥ 35.0kg/m²) (N = 866)** | |  |  |  |  |  |  |  |  |
|  | Decrease (Delta BMI < 0 kg/m²) | 0.95 (0.61, 1.46) | 0.81 |  | 0.93 (0.58, 1.52) | 0.78 |  | 0.73 (0.41, 1.32) | 0.30 |
|  | No change (Delta BMI = 0 kg/m²) | Ref | |  | Ref | |  | Ref | |
|  | Increase (Delta BMI > 0 kg/m²) | 0.99 (0.64, 1.52) | 0.96 |  | 1.03 (0.63, 1.68) | 0.91 |  | 0.98 (0.54, 1.75) | 0.94 |

*Abbreviation: BMI, Body Mass Index; CI, Confidence Interval; OR, Odds Ratio R-SES, Rosenberg Self-Esteem Scale*

*^1^ model 3: adjusted on age, gender, educational level, occupational status, monthly household income, smoking status, physical activity, energy intake and diet quality (mPNNS-GS,* *modified French National Nutrition and Health Program Guideline Score).*

*^2^ model 4: adjusted on age, gender, educational level, occupational status, monthly household income, smoking status, physical activity, energy intake and depressive symptomatology (CES-D, Center for Epidemiology Studies-Depression).*

*^3^ model 5: adjusted on age, gender, educational level, occupational status, monthly household income, smoking status, physical activity, energy intake and anxiety (STAI-T, State--Trait Anxiety Inventory).*

*^4^ P-Value based on multinomial logistic regression with baseline self-esteem as a continuous independent variable and delta BMI as a categorical dependent variable*
